# Free Medical Schools

**Published:** 1850-05

**Authors:** 


### Part 4.—Editorial.

### ARTICLE I.

#### FREE MEDICAL SCHOOLS.

MESSRS EDITORS:—

I find in the February number of the Western Lancet, some remarks in reply to my observations on Free Medical Schools, contained in the January number of your Journal. The only part of the article, however, which bears any semblance of argument, is embraced in the two following paragraphs.—"Professor Davis refers to free teaching in France, and intimates that it has resulted in elevating the profession of that country, and supposes that the same result would follow here. The illustration is singularly inapt; for in France, the most rigid laws exist, prohibiting irregular practice. If each state, (or so many as have suitable localities,) would endow medical colleges, and then by legislative enactment prohibit all from practicing who were not graduates of those colleges, the plan might become beneficial; but as such restrictions can never be secured, it is altogether absurd to discuss such contingencies."

"It is estimated by Prof. D. that this system would induce pupils to study longer, and consequently more accurately. Our experience teaches the contrary. Most, if not all schools, admit pupils free to a third course, and yet how small a proportion avail themselves of this system? Not one in fifty! They are not even willing to pay the expenses of board, for the privilege of attending three or more courses."

The editor of the Ohio Medical and Surgical Journal, quotes these paragraphs, and rather *naively* adds:—"We leave Prof. Davis, to make a satisfactory reply to these strictures as best he may."

If we scan these propositions a little, we shall find them to contain the following elements, viz;—First; in France where the most stringent laws prohibit all irregular practice, it does very well to make medical schools altogether free. Second; if the same prohibitory laws existed and were enforced in this country, the same system of schools "might become beneficial." But as such restrictions on irregular practice, do not exist here, and cannot be enacted, nothing but the most disastrous results could follow from making *medical tuition free*. In other words, whenever regularly educated medical men, are fully protected by law, and enjoy an entire monopoly of medical business, it is perfectly right and beneficial to give them their education gratuitously, that is, in *Free Medical Schools.*

But whenever they are left entirely unprotected, to compete with all the forms of quackery that human ingenuity can invent; and where every man can be a *doctor* who chooses, it is absolutely necessary that the few who would be educated, should be made to pay well for their knowledge. I am certain that no one but a Philosopher, could have broached such a doctrine; for a man of plain common sense would certainly have reversed the whole, and said that when the profession was effectually protected from all irregular competition, its members could well afford to pay high fees for tuition in colleges—while in those countries where every one is allowed to practice as he pleases, college-fees should be reduced to the lowest point, or entirely abolished; for the purpose of inducing as large a proportion of those who are determined to practice, to become educated, as possible.—We have often heard of parents, who expose their children to inclemency of weather and other hardships, for the purpose of making them *tough* and *hardy*; but we are certainly indebted to our friend of Cincinnati, for the idea, that the educated portion of our profession are rendered better able to protect themselves, and compete successfully with unrestricted quackery, by pretty thoroughly exhausting their pockets during the period of their pupilage. The truth is, that wherever all unlicensed practice is effectually prohibited by law, the exacting of high fees by medical colleges, would truly tend to limit the number of practitioners, and thereby lessen competition in practice.

But when, as in this country, no prohibition whatever exists, every man or woman being allowed to dabble in medicine who choses; the maintainance of high charges can have no other possible effect than to limit the number who become educated, and increase in the same proportion the number of the uneducated; while the lower you make these charges, the greater will be the proportionate number who will be induced to prepare themselves properly for their calling. Hence, if our allusion to France as an illustration of the beneficial effects of free medical schools, was in anywise *inapt*, that inaptness was entirely in the opposite direction from that indicated by the Lancet.

The second proposition which we have quoted from the Lancet, main tains that low college fees would not induce students to study longer, because *experience* has shown that very few will consent to pay their board, to attend a *third* course, which is now offered by all the schools *gratuitously.* If the Editor of the Lancet had stated, that the present expenses of gaining a regular medical education, by attending two full courses of lectures, were such, that they completely exhausted the pecuniary means of four-fifths of the students in the country, and caused many of them to pledge their *credit*, for money enough to purchase a few books, a pocket-case of instruments, and medicine enough to begin the work of prescribing for the sick; and that the offer of a *third* course gratuitously, was, to the great mass of students, little better than a mockery, he would have stated a *fact*.

But does this prove that diminishing the expenses of the first two courses, one half, would constitute no inducement for the student to attend a *third*? By no means!—for both reason and experience teach the contrary. The *embellishments* which the Editor of the Lancet has introduced into his article, in the way of talk about inconsistenc es, "a *faux pas*," and "*ad captandum schemes*," we are not disposed to meddle with. I will correct one error, however, which seems to have given both him and the Editor of the Ohio Journal, some trouble. It consists in the statement, that while some of my *wealthy* colleagues are teaching for nothing, *I* am receiving the usual fee, viz: "*Ten dollars per ticket.*" I would state for the satisfaction of all concerned, that I receive *no more* for teaching, than the most wealthy of my colleagues. And if my highly esteemed friends at Cincinnati and Columbus, will think a little, they will doubtless see how *three tickets* can be given gratuitously to a class of students, and yet not have it follow, as a *logical necessity*, that three professors should lecture for nothing. I am gratified, however, to learn that our friend of the Lancet, "does *not* advocate exorbitant fees; on the contrary, *he* believes that even *those in their own school might be reduced a little.*" Certainly there is still hope for the cause of truth and sound policy. N. S. D.

CHICAGO, April 8th, 1850.

